# A case of Warthin-like papillary thyroid carcinoma with diffuse sclerosing stroma and a novel RET mutation: a new entity or a combined tumour?

**DOI:** 10.3332/ecancer.2019.965

**Published:** 2019-10-02

**Authors:** Fausto Maffini, Daniele Lorenzini, Daniela Lepanto, Elvio De Fiori, Caterina Fumagalli, Alessandra Rappa, Marta Tagliabue, Massimo Barberis

**Affiliations:** 1 Department of Pathology, European Institute of Oncology, Via Ripamonti 435, 20141 Milan, Italy; 2 University of Milan School of Medicine, Via Ripamonti 435, 20141 Milan, Italy; 3 Department of Radiology, European Institute of Oncology, Via Ripamonti 435, 20141 Milan, Italy; 4 Division of Otolaryngology and Head and Neck Surgery, European Institute of Oncology, Via Ripamonti 435, 20141 Milan, Italy; * These authors contributed equally to writing this article.

**Keywords:** RET, BRAF, thyroid carcinoma, mutation, pathology

## Abstract

Activating mutations of the *RET* gene have been described for both papillary (chromosomal rearrangement) and medullary (missense mutations) thyroid carcinomas. Here, we describe a case of a Warthin-like variant of papillary thyroid carcinoma displaying some morphological aspects that mimic the diffuse sclerosing variant. The tumour harboured *BRAF* V600E mutation and a novel germline point mutation in the *RET* gene, with unknown clinical and pathological meaning.

## Introduction

Papillary thyroid carcinoma (PTC) is the most common type of thyroid malignant tumours, accounting for up to 80% of overall cases [1]. Although most of them belong to the so-called classic phenotype, several variants have been described and currently classified according to the WHO classification [2]. In particular, a group of these variants features oncocytic changes in the cytoplasm of neoplastic cells and includes the Warthin-like variant (WL-PTC), first described by Apel *et al* [3]. This variant derives its name from the close morphological resemblance to the homonym major salivary glands tumour. It exhibits an architectural pattern constituted of papillary fibrovascular stalks lined by columnar-cuboidal oncocytic neoplastic cells, with nuclear features typical of PTC (i.e., nuclear enlargement, chromatin clearing, inclusions and grooves), and a prominent lymphocytic infiltrate surrounding them. This particular tumour is almost always associated with chronic autoimmune thyroiditis in the non-neoplastic gland [4]. According to current knowledge, this variant does not bear any worse prognostic meaning when compared with classic type with concurrent autoimmune thyroiditis, in contrast with the ‘pure’ oncocytic variant of PTC. It has been hypothesised that its excellent prognosis could be related to Hashimoto thyroiditis [5].

In the past decade, activating mutations of BRAF (a proto-oncogene serine/threonine kinase), in particular, those involving V600E exchange, have been detected in a high percentage of PTC and the clinical relevance of this finding is still under debate. In fact, the presence of *BRAF* mutation is a marker of an aggressive neoplasm, but it is not related to any increase in mortality and recurrence rates [6].

The second most common molecular alteration found in PTC is one of thirteen different chromosomal translocations involving the RET proto-oncogene, yielding various fusion proteins referred to as RET/PTC, demonstrated by Grieco *et al* in 1990 [7]. These rearrangements imply constitutional dimerisation and consequent activation of RET tyrosine-kinase domain, followed by an increase in cell proliferation [8]. A recent study showed that the prevalence of *BRAF* mutation was higher in WL-PTC (26/40) than in the conventional variant, in accordance with the results of previous studies conducted in smaller case series [5, 9, 10]. However, this difference disappears when data are normalised for autoimmune thyroiditis [5].

On the other hand, only two studies investigated the state of RET oncogene in four cases of WL-PTC, using immunohistochemistry, with three tumours harbouring one of the previously described translocations [11, 12].

## Case report

In May 2017, a 34-year-old female presented with a nodule in the right thyroid lobe. The nodule was diagnosed as malignant upon cytological examination performed in another centre and classified Tir-5, according to the Italian Thyroid Classification (Category 6-Malignant according to the Bethesda system for reporting thyroid cytopathology) [13, 14].

The patient was affected by longstanding autoimmune thyroiditis treated with oral levothyroxine 100 μg/day.

**Figure 1.**
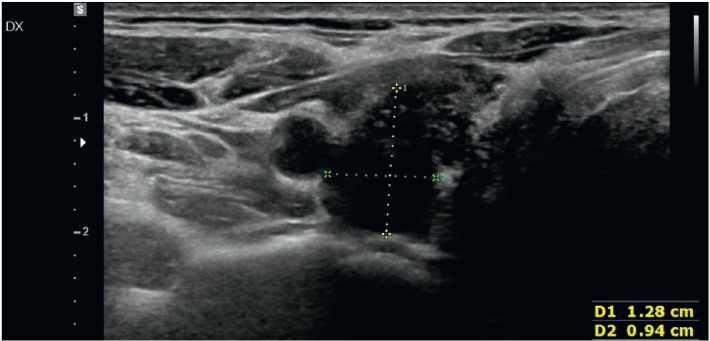
US image showing a hypo-echoic nodule in the right lobe, with a long-axis diameter of 1.3 cm, without peripheral halo and with vascular pattern in the inner area.

The ultrasound (US) examination of the thyroid, performed in our institution, showed parenchymal disarray with a hypo-echoic nodule in the upper third of the right lobe, with a long-axis diameter of 1.3 cm, without peripheral halo nor increased vascular pattern in its inner area (Figure 1).

Since the clinical staging was cT1b cN0 (TNM 7th ed) [15], the patient underwent complete thyroidectomy (justified by her chronic autoimmune thyroiditis already in hormone-replacement therapy) and central neck lymph node dissection; all the four areas were resected (ABCD) following the institutional guidelines [16].

Surgical specimens were fixed in buffered formalin for 24 hours, and at gross examination, we identified an ill-defined hard fibrous area, with a haemorrhagic focus, in the upper third of the right lobe.

Consequently, selected specimens of thyroid parenchyma were paraffin-embedded, cut in 5-μm slides and stained with standard Haematoxylin and Eosin stain (see Appendix: Supplementary Figures).

**Figure 2.**
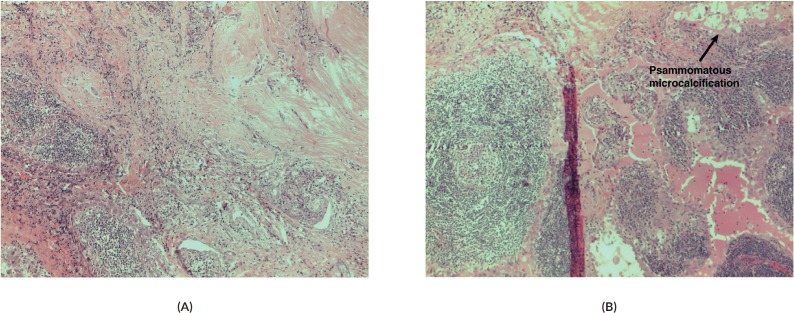
(A) Papillary carcinoma sclerosing variant x200 magnification. (B) Papillary carcinoma sclerosing variant upper left merged with Warthin-like areas; in the upper right psammomatous, microcalcifications are clearly evident x200 magnification.

The fibrous ill-defined area corresponded to the carcinoma, with two different histological patterns: one constituted by a dense fibrous area with slender neoplastic cells, with PTC cytological features and foci of squamous metaplasia, intermingled with lymphoid cells; the second composed by broad papillary fronds encircled by columnar-cuboidal cells with a dense lymphoid infiltrate, with scattered germinal centres. Also, neoplastic cells in this area showed the cytological features of PTC. While the last area resembled a WTL-PTC (Warthin Like-Papillary Thyroid Carcinoma) (Figure 2A), the first component was similar to the diffuse sclerosing variant of PTC (Figure 2B). All identified lymph nodes were negative for metastatic tumour.

Given the different prognostic meaning of the Warthin-like and the diffuse sclerosing variants, we performed an immunohistochemical evaluation of cytokeratin 19, Galectin-3 and p40 expression according to manufacturer’s instructions (all markers were purchased from Dako, Dakopatts, Denmark).

**Figure 3.**
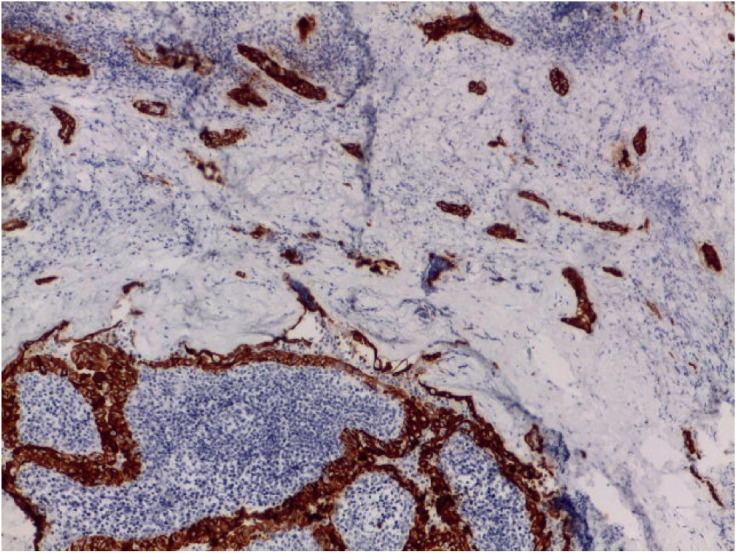
Intense cytoplasmatic Galectine-3 immunostaining in both Warthin-like and sclerosing areas.

**Figure 4.**
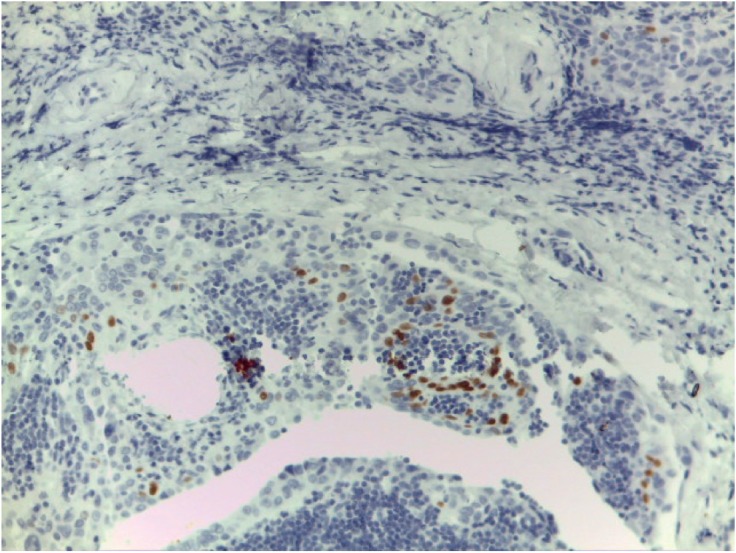
Nuclear p40 immunostaining consistent with squamous differentiation in few neoplastic cells in sclerosing areas.

We detected an intense immunoreactivity for cytokeratin 19 and Galectin-3 (Figure 3) in every neoplastic cell and a focal positivity for p40 in few neoplastic cells in sclerosing areas (Figure 4). Moreover, we investigated *BRAF* and *RET* mutational status of the neoplastic population by next-generation sequencing (NGS) technology, applying the Oncomine Focus Assay (Thermo Fisher, Waltham, MA) according to the manufacturer’s instructions.

**Figure 5.**
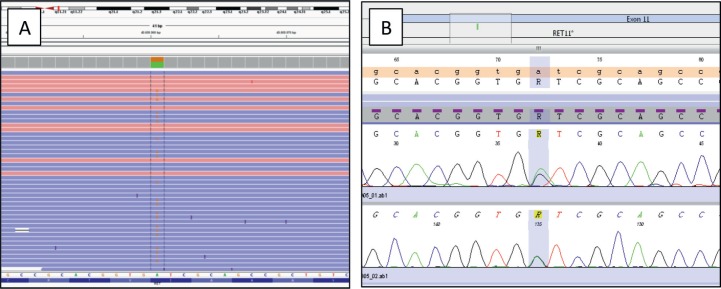
(A): IGV (Integrative Genomics Viewer) graphic showing a single nucleotide variant (SNV) in the RET gene (A > G), producing p.Ile638Val alteration. Forward and reverse strands are displayed in different colours. The reference sequence is reported below (NM_020975.4). (B): Electropherogram obtained by Sanger sequencing confirming the p.Ile638Val mutation.

The most representative areas of both neoplastic components (about 1:1 ratio) were selected and were manually macrodissected before nucleic acid isolation (tumoural cell content after enrichment equal to 20%). Genomic DNA and RNA were extracted using the Qiagen AllPrep FFPE (Formalin Fixed and Paraffin Embedded) DNA/RNA Kit (Qiagen, Valencia, CA) and then quantified with the Qubit high-sensitivity DNA/RNA assay (Life Technologies, Carlsbad, CA). Libraries were constructed starting from 10 ng of DNA/RNA and templates were prepared using the Ion Chef Machine (Thermo Fisher, Waltham, MA). Sequencing of templates was performed on an Ion Torrent S5 Sequencer (Thermo Fisher, Waltham, MA) and the data were analysed using the Ion Reporter variant caller software v.5.6. The NGS analysis revealed a point mutation in exon 15 of *BRAF* (p.Val600Glu, p.V600E) with an allele frequency of 5% (coverage 1,995 reads) and a point mutation in exon 11 of *RET* (p.Ile638Val, p.I638V) (coverage 2,000 reads) with an allele frequency of 47%, never described before to the best of our knowledge. We additionally tested the corresponding normal thyroid tissue and found the same RET variant, (Variant allele frequency = 48%), confirming the germinal origin of the variant. Both variants were confirmed by real-time PCR and Sanger sequencing, respectively, according to Mu *et al* [17]. No *RET*-fusion genes were identified by NGS (Figure 5). The patient was discharged after 4 days without any complications, in treatment with hormone replacement suppressive therapy (0.1 < TSH (Thyroid-Stimulating Hormone) > 0.5); she was free of disease at last follow-up.

## Discussion

This case was challenging due to the differential diagnosis between the Warthin-like and the diffuse sclerosing variants of PTC.

The prognosis of these two variants is different: the former has a good prognosis, comparable to the classic type of PTC, while the latter is characterised by a higher incidence of cervical nodes involvement, reported as around 80%, and distant metastases, present in 10%–15% of cases [18]. These findings correlate with an increased risk of neoplastic relapse and mortality in patients with diffuse sclerosing variant of PTC, as compared with classic variant, although very few studies have so far recorded overall survival in diffuse sclerosing variant [19].

In the present case, we observed a thick fibrous stroma encircling neoplastic cells, associated with psammoma bodies, squamous metaplasia (with p40 positive neoplastic cells) and a dense lymphocytic infiltrate; the histological and immunohistochemical evaluation alone was insufficient to achieve the correct diagnosis.

The analysis of *BRAF* mutation and *RET* rearrangement has been useful to discern between these two variants. In fact, *BRAF* activating mutations have been reported in the literature in a high percentage of WL-PTC [5]; the existence of various substitutions involving V600 codon of the BRAF protein underscores the importance of a complete evaluation of the mutational status of this gene [12]. Moreover, we found a 5% allele frequency of *BRAF* mutation; such a low value may be explained because of other non-neoplastic cells commixed with neoplastic ones (i.e., lymphocytes, stromal cells), highlighting the clinical relevance of using a high-sensitivity technique, such as next-generation sequencing (tumoural cell content of specimens tested: 20%). Recently, some authors identified a genetic alteration of the MAPK (Mitogen-Activating Protein Kinase) pathway in about 70% of PTC using NGS, detecting either point mutations or structural rearrangements [20]. This technique allowed the recognition of other novel molecular alteration in PTC, as in our case [21].

This finding helped us in the differential diagnosis, *BRAF* mutation has rarely been reported in diffuse sclerosing variant and this variant is frequently characterised by RET-PTC rearrangement, which we did not find in our case [22, 23]. It has been hypothesised that the aggressive behaviour of diffuse sclerosing variant is linked to its molecular peculiarity.

Globally, morphology, immunohistochemistry (IHC) and molecular results, involving *BRAF* and *RET* genes, led us to the correct diagnosis.

As unexpected result following NGS, we observed a new mutation involving exon 11 p.Ile638Val (c.1912A > G) of RET that had not been reported before to the best of our knowledge. This mutation was observed also in non-neoplastic thyroid tissue, representing a germline alteration.

In medullary carcinomas, RET is often activated by missense mutation (95% of familiar cases and 40% of sporadic ones), while in PTC, the activation is due to chromosomal rearrangements [24]. The hotspot regions involved are exon 11 (codon 634) and exon 16 (codon 918), frequently mutated in multiple endocrine neoplasm (MEN) syndromes and in sporadic medullary carcinomas [25]. However, isolated mutations have been identified in other codons, such as codons 639, 641 and 922, reported by Kalinin and co-workers [26]. Due to the rare occurrence of PTC in patients affected by MEN2, it has been suggested that activating *RET* point mutations could play a role in the pathogenesis of this neoplasia [27]. Missense mutations have never been described in sporadic cases at the best of our knowledge.

The mutation we observed has not been previously reported and the clinicopathological significance of this finding is still unknown. We can speculate that this *RET* variant could be potentially pathogenic because the amino acid change involves the transmembrane domain of the protein, encoded by codons 636–657. Indeed, other mutations involving this domain have been previously associated with an alteration in hydrophobicity causing a conformational change and an increase in RET kinase activity [28]. Moreover, the in-silico tool ‘PolyPhen2’ predicts that the p.Ile638Val variant is potentially deleterious for RET protein functionality [29]. We speculate that the presence of this double genetic alteration could be responsible for the peculiar histopathological features of the neoplasm; we cannot exclude that patient’s neoplastic risk is increased due to that germline mutation since it has not been described before.

Further studies in larger case series are warranted in order to identify the clinical and pathological implications of this novel mutation. In parallel, the molecular changes caused by this amino acidic substitution remain to be further characterised and could account for possible pathogenic effects, for instance, by constitutional dimerisation or activation of the kinase domain.

## Conclusion

This is a rare case characterised by double morphological features, Warthin-like and diffuse sclerosing-like, with a different prognosis and with a new mutation never described before to the best of our knowledge. We speculated if it were a collision tumour or only a neoplasm with double differentiation and uncertain prognosis.

## Conflicts of interest

The authors declare that they have no conflicts of interest.

## Funding

None of the authors and technicians received funds or funding for the realization of the article.

## Figures and Tables

**Figure S1.**
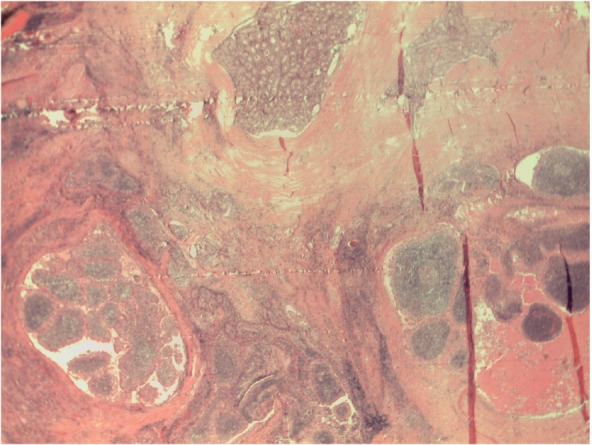
Landscape of papillary carcinoma with two components: bottom left the Warthin-like and upper middle-right the sclerosing ones.

**Figure S2.**
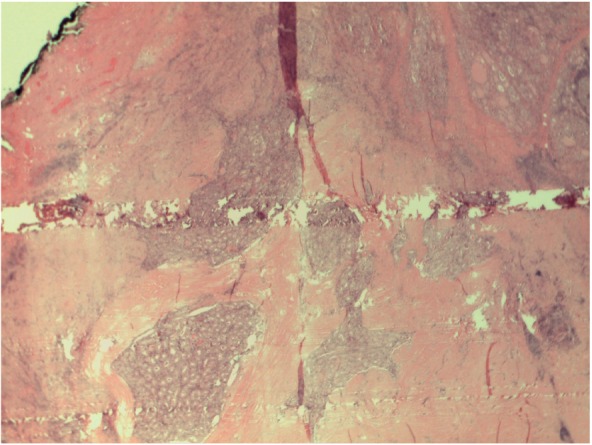
The sclerosing variant of papillary carcinoma with inflammation, sclerosis.

**Figure S3.**
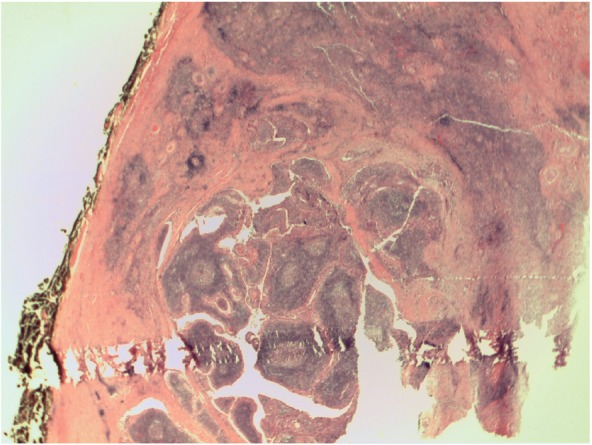
Papillary carcinoma another Warthin-like features.

**Figure S4.**
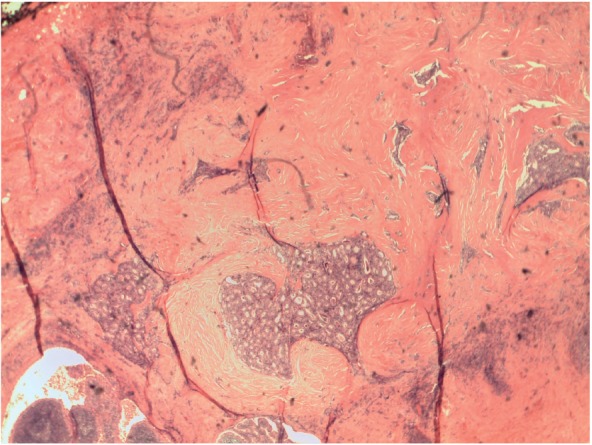
Papillary carcinoma sclerosing variant.

**Figure S5.**
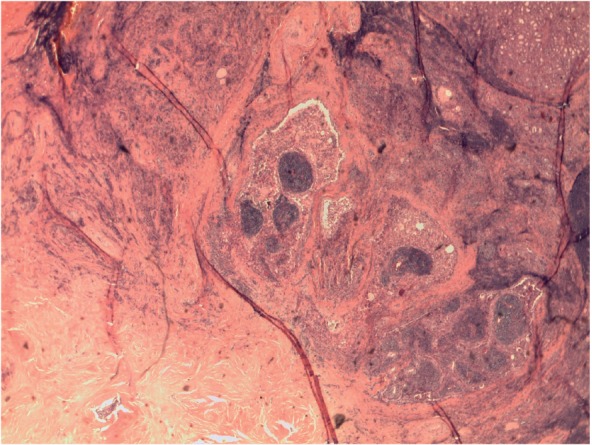
Papillary carcinoma Warthin-like merging with sclerosing ones.

**Figure S6.**
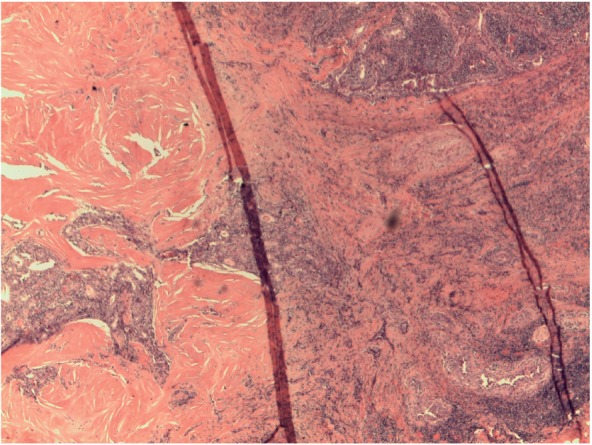
Papillary carcinoma with only sclerosing areas.

**Figure S7.**
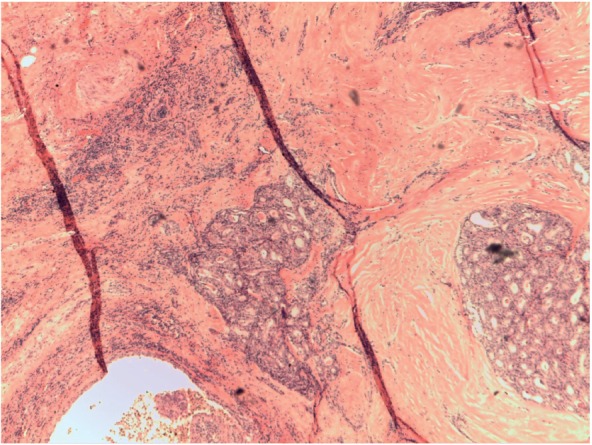
Papillary carcinoma with sclerosing X10 magnification.

**Figure S8.**
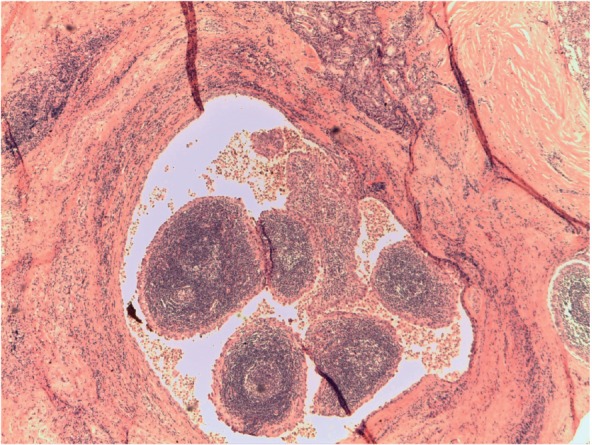
Papillary carcinoma Warthin-like x100 magnification.

**Figure S9.**
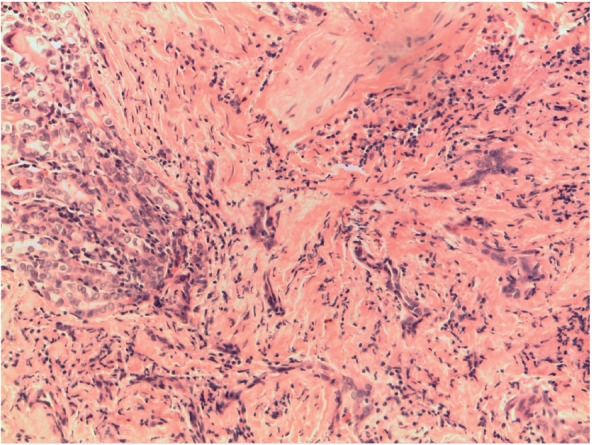
Papillary carcinoma sclerosing variant with inflammation and desmoplasia. X400 magnification.

**Figure S10.**
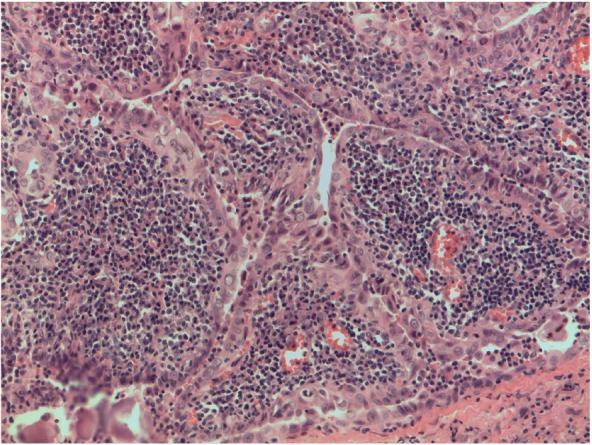
Papillary carcinoma Warthin-like with classical features of Warthin tumor; papillary growth, lymphocytes rich stroma and thyrocytes with papillary nuclear features. X400 magnification.

**Figure S11.**
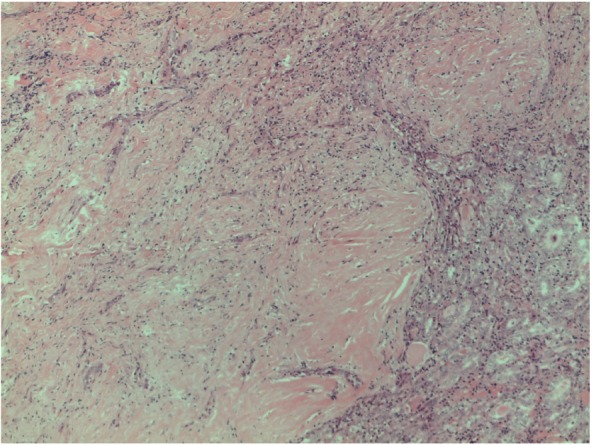
Papillary carcinoma sclerosing variant x200 magnification.

**Figure S12.**
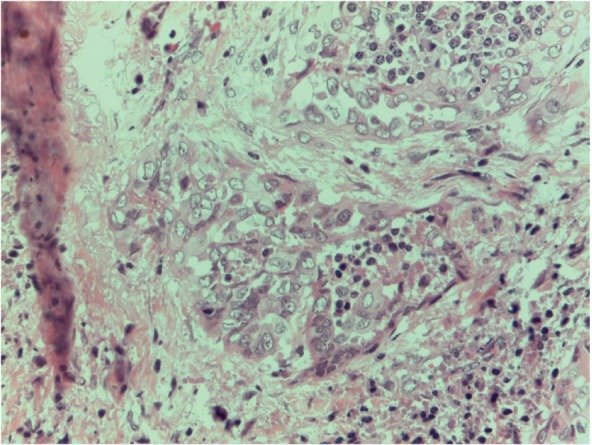
Papillary carcinoma sclerosing variant x400 magnification.

**Figure S13.**
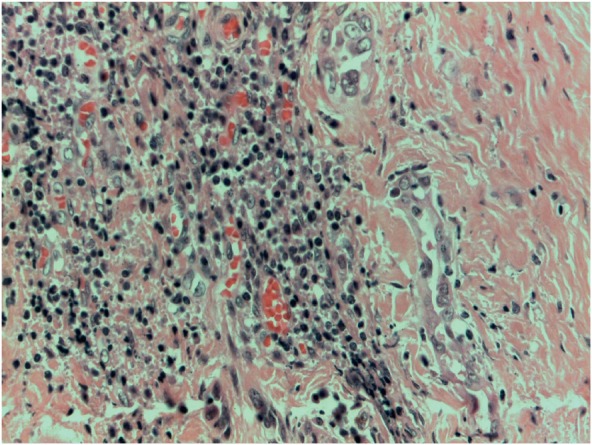
Papillary carcinoma sclerosing variant x400 magnification.

**Figure S14.**
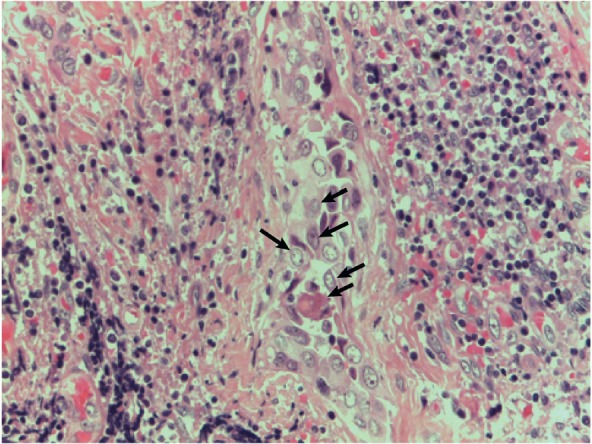
Papillary carcinoma sclerosing variant with the squamous metaplasia area clearly evident by p40 staining and indicated by arrows. x400 magnification.

**Figure S15.**
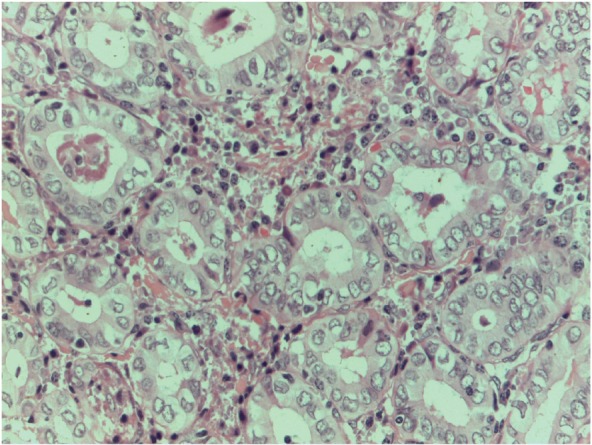
Papillary carcinoma cytoarchitectural features particular x400 magnification.

**Figure S16.**
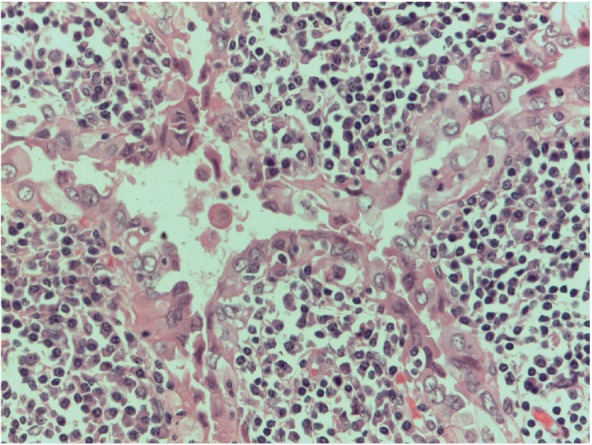
Papillary carcinoma Warthin-like cytoarchitectural features particular x400 magnification.

**Figure S17.**
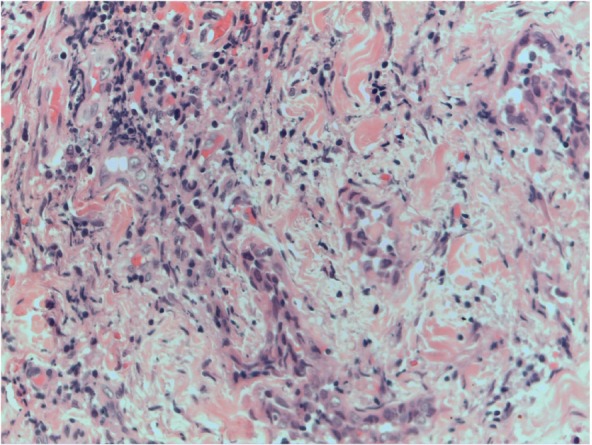
Papillary carcinoma sclerosing variant cytoarchitectural features x400 magnification.

**Figure S18.**
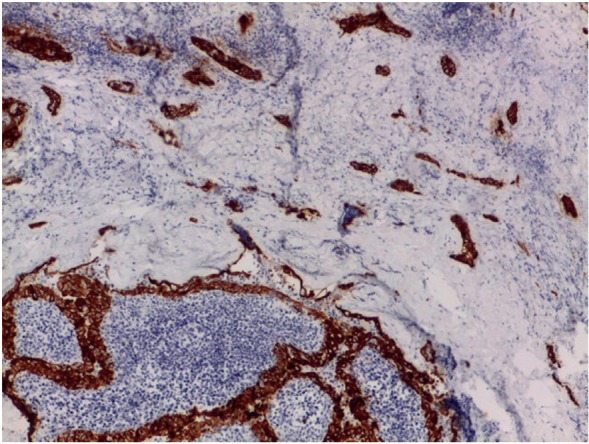
Galectin-3 expression in both areas of Warthin-like tumor and sclerosing ones (Brown staining).

**Figure S19.**
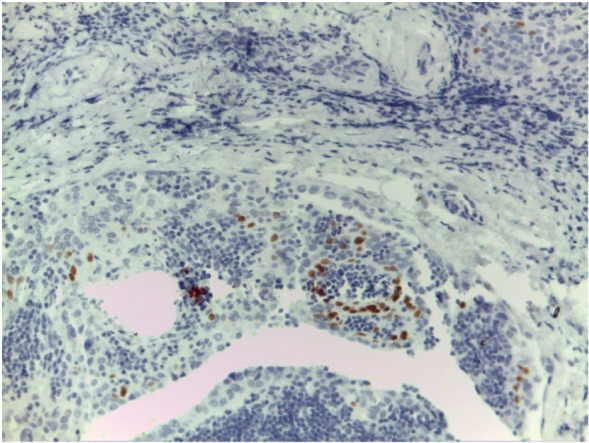
Squamous metaplasia highlighted by p40 nuclear immunostaining (Brown staining).
